# Supplementary material for: Impact of Dextran-Sodium-Sulfate-Induced Enteritis on Murine Cytomegalovirus Reactivation
Source: Viruses. 2022 Nov 22;14(12):2595. doi: 10.3390/v14122595 (PMC9781000; doi:10.3390/v14122595)
Supplement: Supplementary file 1 [file viruses-14-02595-s001.zip › viruses-2007919-supplementary.pdf]

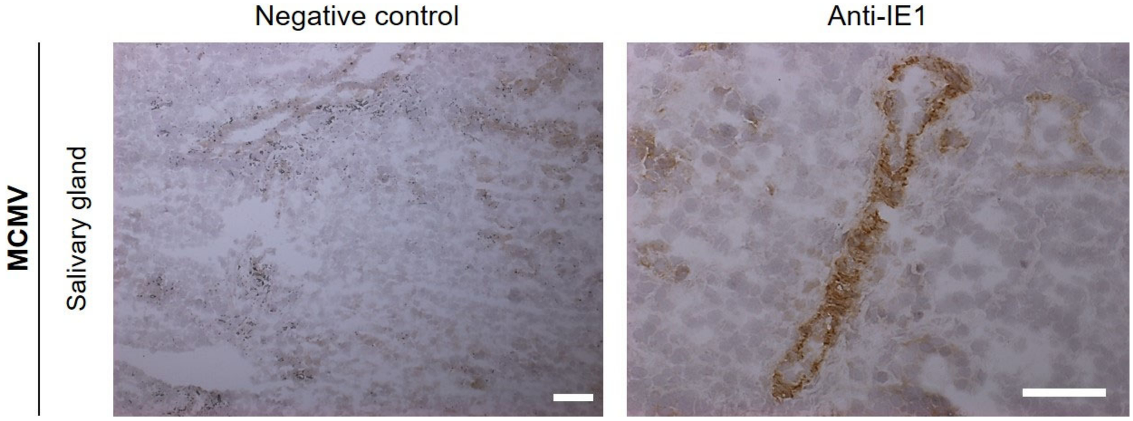

Supplementary Figure S1: Positive MCMV labelling of mouse salivary gland

Immunohistochemistry at D21 (day of sacrifice) on salivary glands specimens of infected mice with 3×10^3 plaque-forming units (PFU) administered by intraperitoneal (IP). Brown parts correspond to positive zones marked by horseradish peroxidase and revealed by diaminobenzidine substrate. The negative control was performed by omitting the primary antibody. The presence of replicative virus was looked for by using an anti-immediate early (IE) 1 protein of MCMV as primary antibody. The scale bar on photographs corresponds to 50 µm. Photographs were taken at 20x objective (negative control) and at 40x objective (anti-IE1 labelling) in Zeiss Axioimager Apotome 3 optical microscope with a Zeiss quadriCCD Axiocam camera.
